# Selenium, Selenoproteins and Viral Infection

**DOI:** 10.3390/nu11092101

**Published:** 2019-09-04

**Authors:** Olivia M. Guillin, Caroline Vindry, Théophile Ohlmann, Laurent Chavatte

**Affiliations:** 1 CIRI, Centre International de Recherche en Infectiologie, CIRI, 69007 Lyon, France; 2 Institut National de la Santé et de la Recherche Médicale (INSERM) Unité U1111, 69007 Lyon, France; 3 Ecole Normale Supérieure de Lyon, 69007 Lyon, France; 4 Université Claude Bernard Lyon 1 (UCBL1), 69622 Lyon, France; 5 Unité Mixte de Recherche 5308 (UMR5308), Centre national de la recherche scientifique (CNRS), 69007 Lyon, France

**Keywords:** reactive oxygen species, glutathione peroxidases, thioredoxin reductases, influenza virus, hepatitis C virus, coxsackie virus, human immunodeficiency virus, molluscum contagiosum virus, viral selenoproteins, immunity

## Abstract

Reactive oxygen species (ROS) are frequently produced during viral infections. Generation of these ROS can be both beneficial and detrimental for many cellular functions. When overwhelming the antioxidant defense system, the excess of ROS induces oxidative stress. Viral infections lead to diseases characterized by a broad spectrum of clinical symptoms, with oxidative stress being one of their hallmarks. In many cases, ROS can, in turn, enhance viral replication leading to an amplification loop. Another important parameter for viral replication and pathogenicity is the nutritional status of the host. Viral infection simultaneously increases the demand for micronutrients and causes their loss, which leads to a deficiency that can be compensated by micronutrient supplementation. Among the nutrients implicated in viral infection, selenium (Se) has an important role in antioxidant defense, redox signaling and redox homeostasis. Most of biological activities of selenium is performed through its incorporation as a rare amino acid selenocysteine in the essential family of selenoproteins. Selenium deficiency, which is the main regulator of selenoprotein expression, has been associated with the pathogenicity of several viruses. In addition, several selenoprotein members, including glutathione peroxidases (GPX), thioredoxin reductases (TXNRD) seemed important in different models of viral replication. Finally, the formal identification of viral selenoproteins in the genome of molluscum contagiosum and fowlpox viruses demonstrated the importance of selenoproteins in viral cycle.

## 1. Introduction

Selenium is an essential trace element for mammalian redox biology. Numerous epidemiological studies have revealed an association between selenium deficiencies and the increased risks of developing several pathologies, including cancers, neurogenerative diseases, cardiovascular disorders and infectious diseases [1,2,3,4,5,6,7,8,9,10,11,12,13]. The ability of selenium supplementation to reverse or reduce these risks has been reported in many human or animal models although it remains controversial [14]. Unlike other trace elements that act as cofactors, selenium is covalently bound to organic molecules. Most of the beneficial effects of selenium is due to its incorporation in the form of selenocysteine into an essential group of proteins that are called selenoproteins. Selenocysteine is the 21st proteinogenic amino acid and is encoded by an UGA codon which is normally the signal for termination of protein synthesis [15,16,17,18,19,20,21,22,23]. Selenocysteine is a structural and functional analog of cysteine in which a selenium atom replaces sulfur to confer an enhanced catalytic activity. Amongst the twenty-five selenoprotein genes identified to date, several have important cellular functions in antioxidant defense, cell signaling and redox homeostasis [24]. Within the well characterized selenoproteins we find the following sub-families: Glutathione peroxidase (GPX1–GPX4 and GPX6) that reduce hydrogen and lipid peroxides [25], thioredoxin reductases (TXNRD1–TXNRD3) which are essentials in the homeostasis of thiol systems [26,27,28,29], methionine sulfoxide reductase (MSRB1) [30] and selenoproteins located in the endoplasmic reticulum (DIO2, SELENOF, SELENOK, SELENOM, SELENON, SELENOS and SELENOT) exhibit important functions in protein folding and in the endoplasmic reticulum stress response [31,32,33]. The other half of the selenoproteome remains without a, yet, defined function. Selenoproteins are present in many organelles or cellular compartments, with a specific tissue distribution and sensitivity to selenium level changes. Selenoproteins are therefore important components of antioxidant defense systems maintaining redox homeostasis, which also include catalase (CAT), superoxide dismutase (SOD), glutathione (GSH), vitamin E, carotenoids, and ascorbic acid.

**Figure 1:**
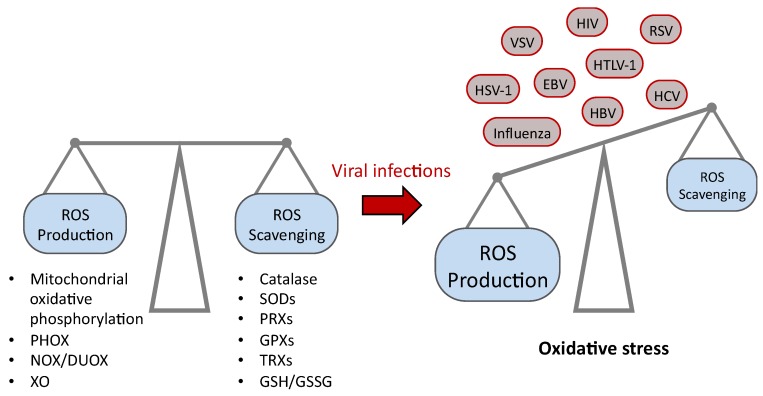
Balance between the generation of reactive oxygen species (ROS) and their scavenging systems in human. This equilibrium can be unbalanced during viral infections, resulting in oxidative stress. The main ROS producing systems include the mitochondrial oxidative phosphorylation, the phagocytic cell NAPDH oxidases (PHOX), the NADPH oxidases/dual oxidases (NOX/DUOX) and the xanthine oxidase (XO). The main ROS scavenging systems include the catalase, the superoxide dismutases (SODs), the peroxiredoxins (PRXs), the glutathione peroxidases (GPXs), the thioredoxins (TRXs) and the balance between reduced and oxidized glutathione (GSH/GSSG). The viruses for which an oxidative stress has been reported are herpes simplex virus type 1 (HSV-1), influenza viruses, vesicular stomatitis virus (VSV), Epstein-Barr virus (EBV), human immunodeficiency virus (HIV), human T cell leukaemia virus type 1 (HTLV-1), hepatitis B virus (HBV), respiratory syncytial virus (RSV) and hepatitis C virus (HCV) [34].

Reactive oxygen species (ROS) are produced during viral infections with both beneficial and deleterious consequences for the cell (Figure 1). The viruses associated with ROS production are human immunodeficiency virus (HIV), hepatitis B virus (HBV), hepatitis C virus (HCV), Epstein-Barr virus (EBV), herpes simplex virus type 1 (HSV-1), vesicular stomatitis virus (VSV), respiratory syncytial virus (RSV), human T cell leukaemia virus type 1 (HTLV-1) and influenza viruses [34]. The mechanisms of ROS generation by the various viruses are diverse, but in several cases the host antioxidant defense enzymes, and especially members of the selenoproteome, are targeted.

## 2. Reactive Oxygen Species (ROS) in Immunity and Viral Infection

### 2.1. ROS and Oxidative Stress

The term “reactive oxygen species” (ROS) refers to series of side-products derived from molecular oxygen (O_2_) generated during mitochondrial oxidative phosphorylation in every respiring cells (Figure 1). ROS can also arise from exogenous sources including drugs, xenobiotics, metals, radiation, smocking and infection [35]. ROS consist of radical and non-radical oxygen species formed by the partial reduction of molecular oxygen. They include superoxide anion radical (O_2_^•−^), hydrogen peroxide (H_2_O_2_), and hydroxyl radical (HO^•^). At low concentration, ROS are also essential molecules in physiological processes such as cell signaling, proliferation, tumor suppression, and maintenance of the immune system. Oxidative stress arises when an imbalance between ROS and the cellular antioxidant defense system occurs (Figure 1). This could be due to an increase in ROS levels or a decrease in the cellular antioxidant capacity. Oxidative stress leads to direct or indirect ROS-mediated damage of nucleic acids, proteins, and lipids, and this phenomenon has been implicated in many pathological conditions including carcinogenesis [36], neurodegeneration [37,38], atherosclerosis, diabetes [39], and aging [40].

The production of ROS can be assessed indirectly either by using redox-sensitive dyes that are oxidized by ROS into quantifiable fluorescent products, such as 20,70-dichlorodihydrofluorescein diacetate (DCFHDA) or by quantification of cellular oxidation products such as oxidized DNA (8-hydroxydeoxyguanosine), lipids (malondialdehyde, F2-isoprostane, 7-ketocholesterol, and 7-hydroxycholesterol), proteins (carbonyl, 4-hydroxynonenal or glycated oxidation products). Many enzymatic assays are also available to evaluate the antioxidant function of the organisms [41].

### 2.2. ROS Function in Immunity and Cell Signaling

ROS have an important role in host defense and immunity [42]. The most characterized example is the mechanism by which phagocytic cells produce large amounts of ROS to eliminate a wide variety of pathogens without altering the host cell viability. The nicotinamide adenine dinucleotide phosphate (NADPH) oxidase enzyme complex of phagocytic cells (PHOX) produces superoxide anion radical in the phagocytic vacuole via the transfer of one electron from NADPH to molecular oxygen [41]. The conventional idea is that this (O_2_^•−^) molecule dismutates to form H_2_O_2_ and other ROS by further chemical or enzymatic reactions [43,44]. Indeed, the myeloperoxidase (MPO) that is an abundant protein released from the granules into the vacuole can further process H_2_O_2_ into HOCl. While the mechanism by which ROS can neutralize the invading micro-organisms in the phagosome is still a matter of debate, the production of HOCl by MPO seems to have a predominant role [43,44].

In addition to microbicidal activity, ROS also act as signaling mediators during cell death/apoptosis but also in processes that control cellular proliferation and differentiation. The family of NADPH oxidases (NOX) and Dual Oxidases (DUOX), referred to as NOX/DUOX, are homologs to PHOX and expressed in a variety of tissues, including colon, kidney, thyroid gland, testis, salivary glands, airways and lymphoid organs. A clear role for cytoplasmic ROS generated by NOX2 as well as DUOX1 has been shown in T cell receptor signaling as well as downstream activation and differentiation of T cells [45,46,47,48]. ROS production by mitochondrial complex III is required for antigen-induced T cell activation and production of interleukin-2 which is the cytokine essential for T cell proliferation [49]. 

### 2.3. ROS and Viral Infection

Viral infection is often accompanied by alteration of intracellular redox state of the host cell [34,41,50,51,52,53,54,55,56,57,58,59,60] (Figure 1). Viruses are known to induce ROS-generating enzymes, including NOX/DUOX and xanthine oxidase (XO) and to disturb antioxidant defenses. XO is implicated in the catabolism of purine nucleic bases by producing H_2_O_2_. Increased of NOX/DUOX and XO activities were observed both *in vitro* and *in vivo* during viral infection [41]. Infection by the HIV is associated with decreased levels of GSH and an increased production of ROS [61,62,63]. The latter can be caused directly by virus and/or by the inflammatory response of the host. The viral TAT protein increases intracellular ROS levels by inhibiting the antioxidant enzyme manganese superoxide dismutase MnSOD [64]. In chronic hepatitis C, direct interaction of core protein with mitochondria is an important cause of the oxidative stress [57]. The increase of ROS production has been well documented during HIV, HBV, HCV, EBV, HSV-1, VSV, RSV, HTLV-1 and Influenza viral infections [34]. With HIV-1, ROS were found to stimulate viral replication with the nuclear transcription factor NF-kB, which is necessary for viral replication, being activated by oxidative stress *in vitro* [54,57,65].

## 3. Selenium, Selenoproteins and ROS

### 3.1. Selenium Insertion in Selenoproteins

Food is the primary source of selenium intake for mammals, but only five molecules (selenocysteine, selenomethionine, selenoneine, selenite, and selenate) constitute the bioavailable selenium in food intake [9,11]. The recommended daily intake of selenium in adults is comprised between 50 and 70 μg per day. A repeated daily intake above 400 μg leads to selenosis and eventually death. However, in certain regions of China, continual intakes of ~1000 µg Se/day are not associated with adverse effects other than fragile hair and fingernails due to keratin disruptions. Importantly, the concentration of selenium measured in the soil and water determines its levels in the living organisms and crops growing in these territories, and notably the components of human food chain, including microorganisms, plants, cereals, vegetables, fruits, farm animals, etc. The importance of selenium as a trace element in human health has been evidenced in a selenium-deprived area of China named Keshan, which then provided the name to the disease, as described in Section 4.1 and in [66]. Strikingly, Keshan disease has been fully eradicated by selenium supplementation [66]. Other regions around the globe are particularly deprived from selenium (<0.1 mg·kg^−1^), and are located in China, New Zealand, Finland, South-East of USA, and in the UK [9].

It is now well admitted that the biological activity of selenium comes from its insertion into selenoproteins as a rare amino acid, selenocysteine [15,16,17,18,19,20,21,22,23]. The human selenoproteome is encoded by 25 selenoprotein genes and is highly regulated by selenium bioavailability [9,10,15,22,67,68,69,70]. Many reports have evidenced a prioritized regulation of the selenoproteome in response to selenium depletion that maintains the expression of essential selenoenzymes at the expense of others. Upon a normal diet, tissue concentration of selenium in the human body ranges from highest levels to lowest: Kidney, liver, spleen, pancreas, heart, brain, lung, bone and skeletal muscle [11]. Interestingly, in animals fed with a low selenium diet, selenium levels are drastically reduced in most tissues, including the normally selenium-rich ones kidneys and liver, but are maintained in very few tissues such as the brain and neuroencrine glands [71]. This phenomenon, described at the scale of organism, tissue, or cell lines, is referred to as selenium or selenoprotein hierarchy [15,22].

The process of selenocysteine insertion relies on a translational mechanism that is unique in many aspects. Selenocysteine was the first addition to the genetic code and is therefore referred to as the 21st amino acid. This amino acid is encoded by the UGA codon, which is normally a stop codon [15,17]. Thus, the cell has evolved a dedicated machinery to recode UGA as selenocysteine in selenoprotein mRNAs while maintaining its use as a stop codon in other cellular mRNAs [15,17]. The selenocysteine insertion sequence (SECIS) located in the 3′ UTR of the mRNA [72] and the selenocysteine-tRNA (Sec-tRNA^[Ser]Sec^) [16], together with their interacting protein partners allow the co-translational incorporation of a selenocysteine amino acid in selenoproteins. This mechanism is rather inefficient (between 1 and 5%), and mostly results in a truncated protein, the UGA codon being read as a stop codon [73,74,75,76,77,78]. Interestingly, more and more reports support the idea that the UGA-selenocysteine recoding event by the ribosome is a limiting stage, and its efficiency dictates selenoprotein expression [15,17,22,67,68,69,70,74].

### 3.2. Role of Glutathione Peroxidases in Antioxidant Defense 

**Figure 2:**
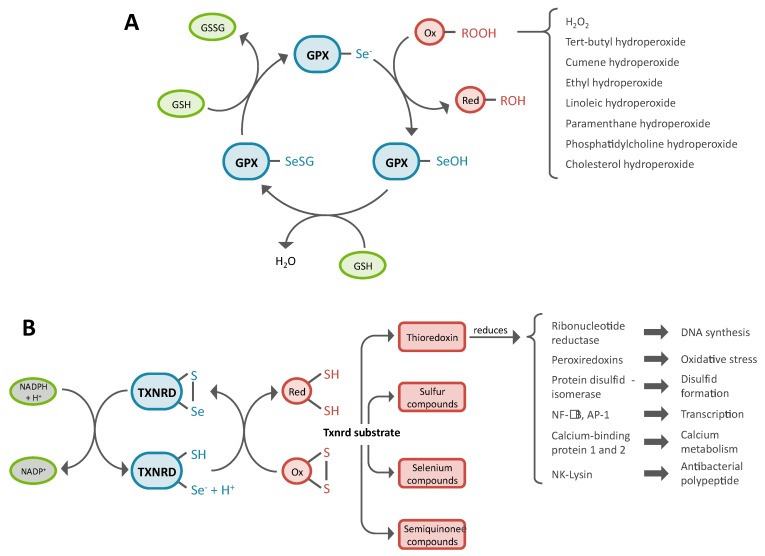
Enzymatic activities of the two most important families of selenoproteins involved in antioxidant defense in mammalian cells: the glutathione peroxidase (GPX) and the thioredoxin reductase (TXNRD). (**A**) GPXs use two molecules of reduced glutathione (GSH) to reduce hydrogen peroxides and organic hydroperoxides (ROOH) in their respective alcohols (ROH). The various peroxide substrates of mammalian GPXs are listed next to the bracket; (**B**) TXNRDs use NADPH to catalyze the reduction of thioredoxins and therefore participate in many cellular functions but can also reduce other sulfur or selenium containing compounds. Ox, oxidized molecule; Red, reduced molecule.

Twenty five selenoprotein genes are present in the human genome [79,80,81], and most of them are involved in a redox reaction [24]. Among the selenoproteome, the GPXs are major components of the mammalian antioxidant defense. In humans, eight GPXs paralogs have been identified, five of them contain a selenocysteine residue in the catalytic site (GPX1–GPX4, GPX6), and three have a cysteine instead (GPX5, GPX7 and GPX8). Among the selenoenzymes, GPX1 and GPX4 are ubiquitously expressed and represent two of the most abundant selenoproteins in mammals. GPX1 is only cytoplasmic while GPX4 is localized in cytoplasmic, mitochondrial, and nuclear cellular compartments. GPX3 is a glycosylated protein secreted in the plasma mostly by the kidney, and its enzymatic activity is commonly used to evaluate the selenium status of the organism as its level fluctuates with selenium intake. GPX2 has initially been described as a gastrointestinal-specific enzyme but is present in other epithelial tissues (lung, skin, liver). Finally, the recently characterized GPX6, is only found in the olfactory epithelium and embryonic tissues. The role of the GPXs is to reduce hydrogen peroxides and organic hydroperoxides before they cause oxidative damage by reacting on cellular components. GPXs use GSH as a cofactor which is subsequently recycled by glutathione reductases [25], Figure 2A. *In vitro*, GPXs are able to reduce a wide variety of substrates that include H_2_O_2_, tert-butyl hydroperoxide, cumene hydroperoxide, ethyl hydroperoxide, linoleic acid hydroperoxide, paramenthane hydroperoxide, phosphatidylcholine hydroperoxide and cholesterol hydroperoxide (Figure 2A). As reported in [82,83] the different GPXs have overlapping enzymatic activities but they exhibit strong substrate specificities. For example, GPX4 is thought to be specialized in the reduction of lipid hydroperoxides while GPX1 is involved in the regulation of H_2_O_2_ metabolism.

The individual role of GPX members has been revealed by gene inactivation in mice. Interestingly, while the inactivation of *Gpx4* gene is embryonically lethal [84], mice deficient in *Gpx1* or *Gpx2* genes are perfectly healthy, fertile and show no increased oxidative stress as compared with wild-type (WT) animals in normal growth conditions [85,86,87]. However, when *Gpx1^-/-^* and WT mice are exposed to lethal doses of pro-oxidant, such as paraquat or H_2_O_2_, an eight-fold decrease in survival is observed for *Gpx1^-/-^* knockout (KO) mice [88,89]. This data suggests that GPX1 has a major role in protecting cells against strong oxidative stress but plays a limited role during normal development and under physiologic conditions. GPX2 appears to have a dual role in cancer, behaving either as a protector of carcinogenesis or a promoter of tumor growth, as revealed by various models [90].

### 3.3. Role of Thioredoxin Reductase in Antioxidant Defense, Redox Homeostasis and Redox Signaling

The two major reductive systems in mammalian cells are the thioredoxin (Txn) and GSH pathways. The Txn system is completely dependent on selenium as the three thioredoxin reductases (TXNRD1–TXNRD3) are selenoproteins with the selenocysteine residue at the penultimate position of the C-terminal end of the protein [26,28]. TXNRD1 and TXNRD2 are ubiquitously present in the cytoplasm and mitochondria, respectively, while TXNRD3 expression is restricted to specific tissues. The primary substrates of TXNRD1 and TXNRD2 are Txn1 and Txn2, respectively, Txn2 being localized in the mitochondria. TXNRDs catalyze the NADPH-dependent reduction of oxidized thioredoxin (Figure 2B). The Txns catalyze the reduction of protein disulfides such as in ribonucleotide reductase, peroxiredoxins (PRX), MSRB1, protein disulfide-isomerase (PDI), and are therefore critical for DNA synthesis, the defense against oxidative stress and disulfide formation within the endoplasmic reticulum [20]. Peroxiredoxins are able to reduce H_2_O_2_, organic hydroperoxides and peroxynitrite in order to protect cellular components from oxidative damage. However, the existence of multiple peroxide-removing enzymes such as catalase, GPX and PRX indicates that these peroxidases are not simply used in oxidant defense [91]. During inflammation, high levels of peroxides are produced by phagocytes to kill microorganisms. It has been well established that PRXs play cytoprotective antioxidant role in inflammation. Recently, it has been proposed that PRXs may play key roles in innate immunity and inflammation [91]. It becomes clear that, in addition to fighting oxidative stress, PRXs are important modulators of peroxide signaling. In addition to Txn, TXNRDs can reduce other small molecules containing sulfur, selenium, or oxidized semiquinone and therefore participate in many other cellular processes [20,26,28,92], Figure 2B.

## 4. Selenium, Selenoproteins and Viral Replication

It is now recognized that the nutritional status of the host plays a leading role in the defense against infectious diseases [93,94,95,96,97]. Many studies show that nutritionally deficient humans or animals are more susceptible to a wide variety of infections. For a long time, researchers have believed that this was only the result of an impaired host immune response due to the deficiency of a particular nutritional element. However, as described below, the mechanism is more complex in that nutritional deficiency impacts not only the immune response of the host but also the viral pathogen itself. Thus, dietary selenium deficiency that causes oxidative stress in the host can alter a viral genome, so that a normally benign or mildly pathogenic virus becomes highly virulent in the deficient host under oxidative stress. This phenomenon has been reported in animal models for influenza and coxsackie viruses [95,98,99], but the molecular mechanism remains unclear. Once the viral mutations occur, even hosts with normal diet would be sensitive to the newly pathogenic strain. The link between selenium levels and viral infection has been reported for many viral groups, see Table 1.

### 4.1. Coxsackie Virus

The coxsackie virus is a nonenveloped, linear, positive-sense single-stranded RNA virus that belongs to the family of *Picornaviridae* (Group IV), genus *Enterovirus*. These enteroviruses, which also include poliovirus and echovirus, are among the most common and important human pathogens [100,101]. Coxsackie viruses are divided into group A (23 serotypes) and group B (six serotypes) viruses. In general, coxsackie viruses from group A infect the skin and mucous membranes, while viruses from group B infect the heart, pleura, pancreas, and liver [100].

In the early 1930s an endemic cardiomyopathy termed Keshan disease was first described in Heilongjiang province, Northeast China. This disease mainly affects infants, children and women in childbearing age [66]. It is characterized by cardiac enlargement, congestive heart failure, pulmonary edema and death. Keshan disease spread in another 12 provinces across China between the 1940s and 1960s. Approximately eight million people lived in the affected areas during that period of time, and thousands of people died of Keshan disease every year from this pathology. It is only in the 1970s and even the early 1980s, that the selenium contents in soil, water, food, and human body fluids were found extremely deficient in the areas affected by Keshan disease as compared with adjacent provinces [102]. Selenium fertilizer was applied to the soil in order to increase its content in the food [66]. In addition, selenium supplementation of the diet was also given to the people of these areas. The result was the complete eradication of this disease in these provinces of China [103]. However, several features of the Keshan disease, especially the annual or seasonal fluctuation in the incidence of the disease, did not wholly fit with a selenium deficiency. It appears that this disease has a dual etiology, i.e., selenium deficiency and an infectious cofactor, namely the coxsackie virus B [103,104,105,106]. 

**Figure 3:**
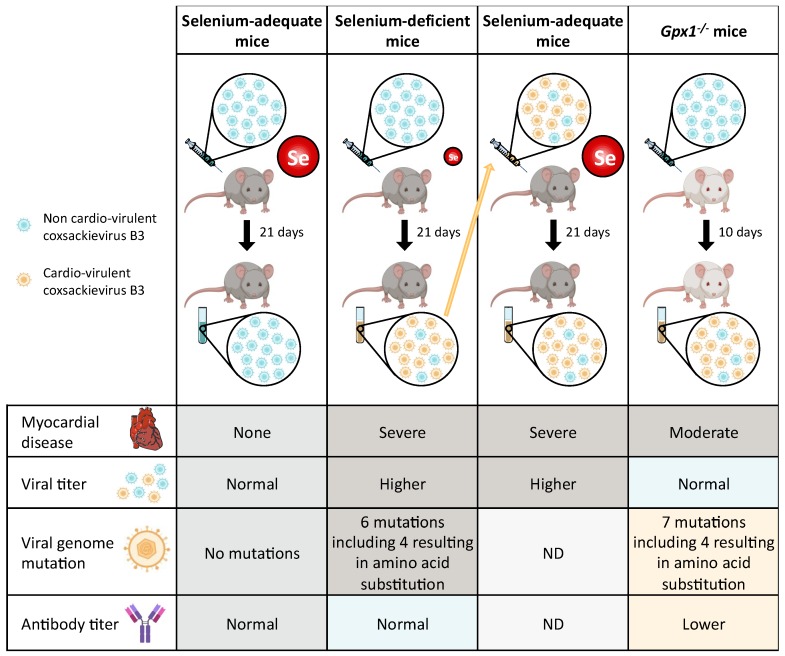
Evolution of the pathogenicity of Coxsackie virus as a function of selenium intake or selenoprotein knockout [53,93,94,96,97,98,99,107,108,109,110,111]. Coxsackie virus B3 (CVB3) infection of mice can cause myocarditis, similarly to that found in human disease. A non-virulent stain of CVB3 (referred to as CVB3/0, and shown in blue) does not lead to myocarditis in this animal model, although replicating in the mice heart fed with adequate selenium diet (left column). In case of selenium deficient mice, a group of animals was fed with a selenium-deficient diet for four weeks before infection with the benign strain CVB3/0 (second column from the left). A control group of animals was fed with an adequate-selenium diet and infected in parallel [98]. In case of selenium-deficient mice, they developed severe myocarditis. The sequencing of the CVB3 viral genome isolated from the heart of selenium-deficient mice showed mutations at nucleotide positions known to co-vary with cardio-virulence of CVB3 strains (shown in yellow). In comparison, the sequence of CVB3 isolated from selenium adequate mice showed no genetic variation (first column). To determine the consequences of the genetic alterations of the virus, CVB3 isolated from selenium deficient mice was inoculated in animals fed with a selenium-adequate diet (third column from the left) [98]. This experiment confirmed that the mutations of the viral genome increased the cardio-virulence of the virus, which can now induce severe myocarditis even in selenium adequate mice. To investigate whether the most abundant selenoprotein, GPX1, which expression correlates with selenium intake, is involved in the virulence of CVB3, a similar study was performed with *Gpx1^-/-^* mice (right column) [108]. These mice, infected with the benign strain CVB3/0, developed myocarditis and nucleotide mutations of the viral genome isolated from their heart, similarly to selenium deficient mice.

Animal models were used to understand the relationship between host selenium nutritional status and coxsackie virus infection [53,93,94,96,97,98,99,107,108,109,110,111,112]. Coxsackie virus B3 (CVB3) infection of mice can cause myocarditis, similar to that found in human populations afflicted with Keshan disease. Interestingly, as illustrated in Figure 3 the work from Beck and co-authors showed that a non-virulent stain of CVB3 (designated CVB3/0) that do not lead to myocarditis, although replicating, is able to evolve in a virulent strain when inoculated in selenium deficient mice [98,99,109,110,111]. Remarkably, this is also true when *Gpx1* knockout mice were infected with the benign strain CVB3/0. The sequencing of the viral genomic RNA isolated from selenium deficient and *Gpx1^-/-^* mice demonstrated that a viral genome change had occurred during the infection and replication of the virus as compared to the viral genome replicated in selenium adequate animals, resulting in a highly pathogenic virus [108]. Out of the ten-nucleotide positions that were reported to co-vary with cardio-virulence in CVB3 strains, six reverted to the virulent genotype in virus isolated in Se-deficient mice, and seven in *Gpx1^-/-^* mice. Interestingly, a similar finding was also reported with the deficiency of another essential antioxidant, namely Vitamin E [93,94]. 

These experiments performed in animal models demonstrate that the host nutritional status, and particularly its antioxidant defense system is an important virulence factor, which can greatly contribute to the evolution of benign viral genomes into more virulent viruses. However, the molecular mechanism involved in this process remains to be elucidated.

### 4.2. Influenza Virus (Orthomyxoviridae)

Influenza viruses are enveloped, linear, negative-sense single-stranded RNA viruses belonging to the *Orthomyxoviridae* family (Group IV). There are four genus of this family: A, B, C and Thogotovirus, but only three influenza viruses are infectious for humans (A, B and C) [113]. The viral genome consists of eight segmented single-stranded RNA segments (seven for influenza C virus) encoding between 9 to 12 proteins, including hemagglutinin (HA) and neuraminidase (NA) surface glycoproteins, three ribonucleic acid (vRNA) polymerase subunits (vRNP: PA, PB1, PB2), non-structural protein (NS1), and matrix proteins M1 and M2 [113]. 

Various subtypes of the most common influenza A viruses are classified based on the diversity in the structure of HA and NA proteins. Influenza viruses can be divided into 16 different HA and NA combinations. Influenza A and B viruses cause epidemics, whereas influenza C virus tends to cause infections with less severe symptoms [113]. According to the World Health Organization (WHO), the seasonal epidemics result every year in 3 to 5 million cases of severe illness and in 250 to 500 thousands deaths worldwide (https://www.who.int/influenza/en/). People at highest risk for mortality are the elderly and individuals with chronic diseases of the lung and heart. However, safe and effective vaccines are available but often do not perfectly match the circulating subtypes or become ineffective due to viral antigenic drift [113]. It is therefore necessary to engineer new vaccines and revaccinate people at risk every year.

The patients infected with influenza virus display a marked increase in DNA, lipid and protein oxidation products in blood plasma and urine [41,114,115,116]. Models of mice and cell lines infected with influenza viruses also show an enhanced production of ROS together with an imbalance of antioxidant defense [117,118,119,120]. These models are relevant to study the changes in redox homeostasis induced by the influenza virus.

**Figure 4:**
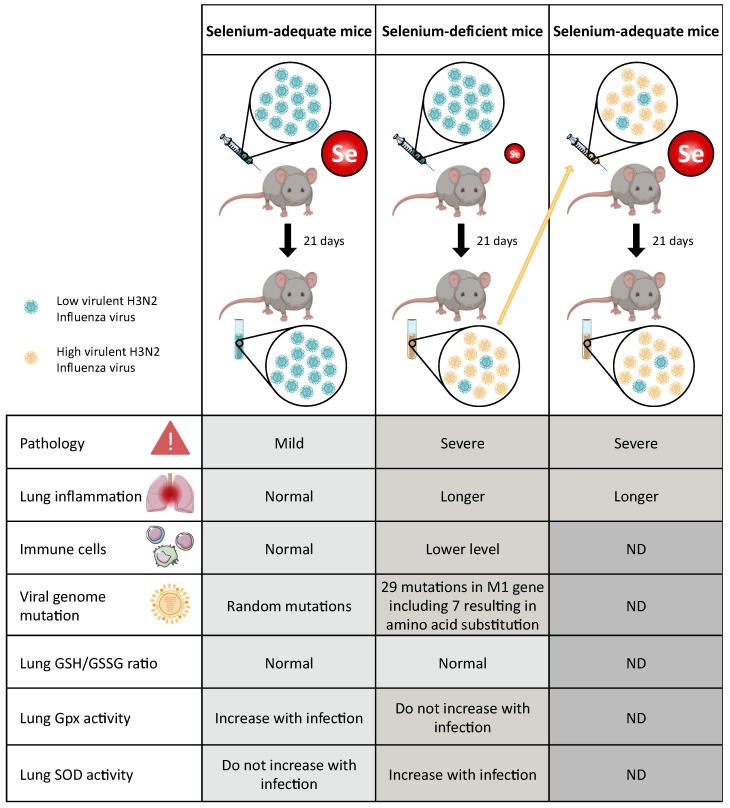
Evolution of the pathogenicity of influenza virus as a function of dietary selenium intake in mice. Influenza A/Bangkok/1/79 (H3N2) virus was inoculated in mice that were previously fed with selenium adequate or deficient diet for four weeks. This virus induces mild pneumonitis in selenium-adequate mice but a severe lung pathology in selenium deficient mice [95,121,122,123,124]. Various parameters, including the time of lung inflammation, the number of immune cells, the nucleotide mutations of the isolated influenza viruses, the oxidation status of glutathione (reduced/oxidized), the GPX and SOD enzymatic activities in the lung, were evaluated and compared between selenium adequate (left column) and deficient (middle column) mice. The low and high virulent H3N2 viruses are represented in blue and yellow respectively. The virus recovered from selenium-deficient mice was inoculated in selenium-adequate mice to evaluate its pathogenicity. Consistent with the observations made with coxsackie virus, the mutations of the influenza viral genome increased the pathogenicity of the virus, which can now induce severe lung pathology even in selenium adequate mice [95,121,122,123,124].

The work from Beck’s laboratory extended this novel concept that host nutritional status (especially selenium deficiency) is an important virulence factor in a viral family other than enteroviruses, as shown in Figure 4 [95,121,122,123,124]. Indeed, a rapid change in the pathogenicity of the virus in selenium deficient host has been also reported for influenza virus similarly to what was found for coxsackie virus. As shown in Figure 4, mice were fed with a diet either deficient or adequate in selenium for 4 weeks. Then, influenza A/Bangkok/1/79 (H3N2), a strain that induces mild pneumonitis in normal mice, was inoculated to both groups of mice. Interestingly, at all-time points post-infection a clear difference in pathology was observed between the two groups of mice [95,121,122,123,124]. The virus was much more virulent in selenium deficient mice, although with a similar virus titer than the selenium adequate mice. In addition, the sequencing of the HA, NA and M genes of viruses isolated from selenium-adequate and selenium-deficient mice demonstrated a strong impact of selenium status on virus mutation.

It appears that the selenium deficiency of the host promotes rapid genomic evolution of the virus in HA and NA genes as compared with selenium adequate animals [53,95,122,123,124,125]. Strikingly, these mutations are not stochastic as they were identical in three independent mice fed in selenium deficient diet. In comparison, very few mutations were detected in animals fed with adequate selenium diet. These data further confirm the impact of selenium status of the host in viral genome evolution. 

### 4.3. Human Immunodeficiency Virus (HIV)

The human immunodeficiency virus (HIV) is an enveloped, linear, positive-sense single-stranded RNA virus that belongs to the family of *Retroviridae* (Group VI), genus *Lentivirus*. Two types of HIV have been characterized: HIV-1 and HIV-2 [126]. Given that HIV-1 is more virulent and more infective than HIV-2, HIV-1 has spread worldwide while HIV-2 is mostly confined to West Africa [127]. HIV is the etiologic agent of acquired immunodeficiency syndrome (AIDS) and is responsible for a weakened immune system as it infects immune cells [126]. HIV affects more than 35 million people worldwide and causes the death of about 1.5 million patients per year (http://www.who.int/hiv/en/). HIV infection is now considered as a chronic disease that requires intensive treatment and can present a variable clinical course. No vaccine is available until now, but an effective medication in decreasing the viral load and increasing the number of CD4 T-lymphocytes has been developed and is referred to highly active antiretroviral therapy (HAART) [128]. This treatment consists in the combination of three or more drugs that target different aspects of HIV replication [129].

HIV genome is highly compact and contains three genes encoding viral structural proteins (*gag*, *pol* and *env*), two genes for essential regulatory elements (*tat* and *rev*) and at least four genes encoding accessory regulatory proteins (*nef*, *vpr*, *vpu* and *vif*). As in any retrovirus, the RNA viral genome is reverse-transcribed in dsDNA that is then integrated in the host genome by the viral integrase. HIV-1 infects immune cells that harbor the CD4 receptor and a co-receptor belonging to the chemokine receptor family (CCR5 and CXCR4) [126]. Therefore, cells infected by HIV-1 are CD4 T-lymphocytes, monocytes, macrophages and dendritic cells. The replication but also the latency of the virus is extremely variable from one cell type to another. 

Lentiviruses are characterized by a long incubation period after the primo infection that is highly variable from one patient to another. During this time, humans infected with HIV are under chronic oxidative stress. The redox status of the patient is strongly disturbed in HIV infected patients as revealed by the decrease of antioxidant defense (selenium, ascorbic acid, alpha-tocopherol, carotenoids, superoxide dismutase, glutathione, and glutathione peroxidase) and the increase in ROS production (hydroperoxides, malondialdehyde, and clastogenic factors) [130]. The altered redox status seems to contribute to HIV pathogenesis in several ways. *In vitro*, increasing oxidative stress enhances the replication of HIV through the activation of NF-kB. Several mechanisms have been reported to explain the cellular enhancement of ROS production in HIV infection. Most of them imply the following viral proteins: Gp120, Tat, Nef, Vpr, and Retrotranscriptase (RT), as reviewed in [50]. A dramatic consequence of this chronic oxidative stress is the fatal decrease in the number of CD4 T-cells by apoptosis, and ultimately a failure of the immune system leading to death. 

The nutritional deficiencies of the HIV-infected patient can affect the responsive capacity of the immune system and the progression to AIDS. Selenium is nowadays understood as an essential micronutrient for antioxidant defense and also immune function [131,132]. HIV infection simultaneously increases the demand for micronutrients and causes their loss which leads to a deficiency that can be compensated by micronutrient supplementation [133,134,135]. Low selenium levels are associated with a lower number of CD4 T-cells, faster progression of AIDS, and 20% increase in the risk of death [133,136]. However, little has been done in term of intervention studies by selenium supplementation or at the cellular and molecular levels to establish the link between selenium, selenoprotein and HIV infection. For example, selenium supplementation is only effective in slowing HIV progression for a subgroup of patients, for which serum selenium levels, CD4 count and viral load were improving in contrast to selenium non-responders or placebo group [137,138,139]. However, the cellular and molecular mechanism for this unequal response remains elusive. Although efficient at controlling viral load and restoring immune function, HIV antiretroviral therapies, especially the protease and reverse transcriptase inhibitors, have been shown to induce oxidative stress [50,140]. Interestingly, a long time treatment (more than 2 years) with antiretroviral therapy improves selenium levels as compared with HIV-infected patients not receiving the treatment [141].

The field awaits further investigations to understand the role of selenium and selenoproteins during HIV infection at the molecular level. The only *in vitro* data available reported a modification of the pattern of selenoprotein expression in response to HIV infection in lymphocytes [142] but these experiments were performed before the complete characterization of the selenoproteome. The impact of selenium status on viral genome mutations and in particular the shift to more virulent viruses has not yet been tested for HIV as it has been done for coxsackie and influenza.

### 4.4. Hepatitis C Virus (HCV)

The hepatitis C virus (HCV) is an enveloped, linear, positive-sense single-stranded RNA virus that belongs to the family of *Flaviviridae* (Groupe IV), genus *Hepacivirus*. Nowadays, about 3% of the world’s population is infected with HCV, which represents approximately 170 million people. Although HCV replication occurs in hepatocytes, the virus also propagates in immune cells. In 80% of the patients with acute hepatitis C, the disease evolves to chronic hepatitis, with 2% developing liver cirrhosis and 1–5% developing liver cancer [143,144]. Many characteristics of oxidative stress have been reported during chronic hepatitis C, including a decrease in GSH, increase in MDA, HNE and caspase activity [145,146]. Zinc and selenium deficiencies increase the risk of chronicity and malignancy [147]. In addition, there is a high prevalence of HCV coinfection in HIV infected patients. The genome of around 9600 nucleotides encodes a unique polyprotein which is co- and post-translationally cleaved into 10 structural and non-structural proteins.

The infection by HCV is another well-documented example of virus-induced generation of ROS. The nucleocapsid protein of HCV, and to a lesser extent NS3, NS5A, E1, E2 and NS4B, are involved in generating oxidative stress in the liver [51,148,149,150,151]. In parallel, the plasma levels of selenium together with erythrocyte GPX activities were significantly lower in HCV-infected patients than in healthy controls. An inverse correlation of selenium levels with viral load was also observed [152]. Interestingly, in HCV and HIV co-infected patients, an even lower serum selenium concentration was measured than in HIV-infected patients [153]. Endoplasmic reticulum stress and unfolded protein response are induced by HCV gene expression [154]. A selenoprotein involved in these mechanisms, SELENOM, has been reported to be upregulated in human hepatocellular carcinoma (HCC) cell lines and liver biopsies of patients with HCV-related cirrhosis [155]. Whether this is true for other endoplasmic reticulum located selenoproteins remains to be investigated.

### 4.5. Other Viruses

The Hepatitis B virus is an enveloped virus with a circular and partially double-stranded DNA that belongs to the *Hepadnaviridae* family (Group VII). HBV includes several viruses that infect liver cells and cause hepatitis in humans and animals. In the viral genome, the large negative stranded DNA encodes the envelope, core and non-structural proteins, the DNA polymerase and an oncogenic transactivator [156,157]. The synthesis of the short strand is completed by cellular DNA polymerases after infection. There are 8 HBV strains, from A to H that differ from their geographic repartition [156,157]. Worldwide, between 2 and 8% of the population is infected by HBV but in most of the cases being asymptomatic. An acute HBV infection is however characterized by yellow eyes and skin, severe fatigue, vomiting and abdominal pain. In less than 5% of the cases, the infected people could develop a chronic infection which can further lead to a cirrhosis (in 20% of the cases) [156,157]. Several studies showed an association link between plasma selenium levels and progression of HBV infection [158,159,160]. For example, the selenium level is not correlated with the responsiveness to interferon treatment [161] but an elevated plasma selenium concentration is associated with a low level of transaminases [161]. Theses hepatic enzymes are implicated in amino acid catabolism, and their release in the plasma is linked to hepatocellular damage. In intervention studies, selenium supplementation decreased cancer incidence in HBV infected patients [162], but when the supplementation was stopped, the incidence became similar to control patients. Finally, *in vitro*, when hepatic cell lines were grown with different selenium concentration, lower viral proteins, viral transcripts and viral genomic DNA were detected with high selenium culture conditions [161].

The Porcine Circovirus 2 (PCV2) is a non-enveloped virus with a circular single-stranded DNA genome which belong to the *Circoviridae* family (Group II) [163]. Two strains exist, type 1 and type 2, but only type 2 causes a disease in swine, namely the Postweaning Multisystemic Wasting Syndrome (PMWS), a dramatic disease for pig-production industry. The severity of this syndrome is thought to highly dependent on intrinsic factor such as the status of the immune system. It is one of the smallest virus characterized so far, encoding only a capsid protein and two necessary proteins for viral replication [163]. It has been shown that selenomethionine supplementation in cell culture inhibits viral replication [164,165,166,167,168]. Furthermore, addition of H_2_O_2_ or ochratoxin A that induced oxidative stress enhanced viral replication. This effect was prevented by selenium supplementation [164,165,166,167,168] or by selenoproteins SELENOS and GPX1 [164,165,166,167,168]. It appears that this mechanism involved the autophagy pathway [164,165,166,167,168]. Finally, in infected mice, selenium supplementation was able to decrease histological lesions by reducing inflammation [164,165,166,167,168].

## 5. Selenoproteins in Viral Genomes

### 5.1. 1998: First Example of a Viral Selenoprotein Encoded in Molluscum Contagiosum Virus Genome

**Figure 5:**
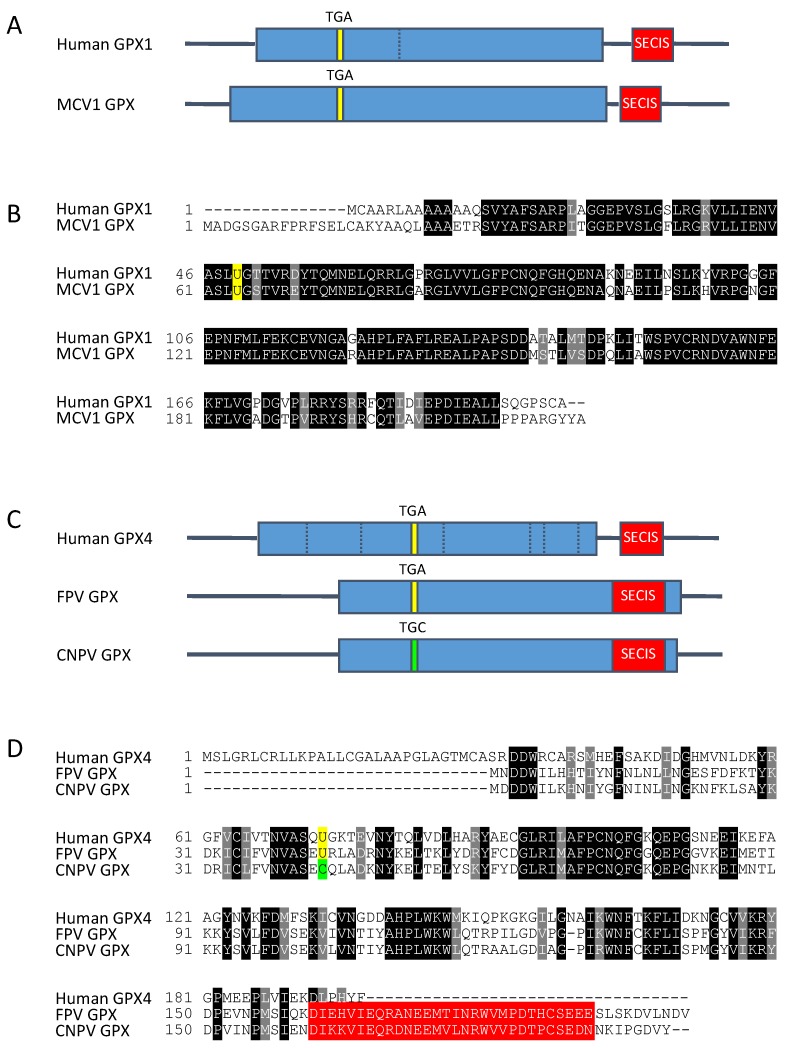
Gene structures and amino acid sequences of the selenoproteins present in the viral genomes of molluscum contagiosum virus subtype 1 (MCV1) (**A**,**B**) and of fowlpox virus (FPV) (**C**,**D**) in comparison with their respective human orthologs, GPX1 and GPX4 [175,176]. (**A**) Location of the typical features of a selenoprotein gene (coding sequence, TGA codon and SECIS element) in human *GPX1* gene in comparison with those of MCV1 *GPX* gene. For clarity reasons, the introns of human gene have been removed, but the position of splice sites is indicated by a dashed bar. (**B**) Amino acid sequence alignment of human GPX1 (P07203) with MCV1 GPX (Q98234). Identical and similar amino acids in both sequences are highlighted in black and grey, respectively. The selenocysteine amino acid (U, in one-letter code) is highlighted in yellow. (**C**) Comparison of the location of selenoprotein gene features in human *GPX4* gene with those of FPV *GPX* and Canarypox virus (CPV) *GPX* genes. The replacement of the TGA (selenocysteine) codon by a TGC (Cysteine) one in CPV *GPX* gene is indicated in green. (**D**) Amino acid sequence alignment of human GPX4 (P36969) with FPV GPX (Q70H87) and CPV GPX (Q6VZR0). In-frame SECIS elements in the C-terminal region of FPV GPX are highlighted in red.

Molluscum contagiosum is a viral infection that affects the skin and is caused by the dermatotropic poxvirus molluscum contagiosum virus (MCV) [237,238]. Unlike smallpox and human monkeypox diseases, MCV is nonlethal, mostly common in children and young adults and present worldwide [237,238]. However, MCV causes severe skin infections in immunosuppressed adults [237,238]. A typical feature is the apparition of single or multiple papules on the skin, which may persist for a few years. Most cases resolve in six to nine months without specific treatments [237,238]. Such a prolonged infection implies that MCV successfully manipulates the host environment. In 1998, the analysis of the MCV genome sequence predicted the presence of a candidate selenoprotein, homologous to mammalian GPX, with 75% amino acid sequence identity with human GPX1 [175], see Figure 5A,B. This viral GPX protein is encoded by *MC066L* gene that presents every features of a selenoprotein gene, i.e., an in-frame UGA codon, a stop codon different from an UGA (in this case UAG), and a SECIS element in the 3’UTR of the mRNA (Figure 5A). The absence of homologs of this gene in vaccinia and variola viruses suggests that the GPX-like gene was acquired by the MCV after the divergence of the *Molluscipoxvirus* and *Orthopoxvirus* genera. The expression of this predicted selenoprotein was tested experimentally in mammalian cells. Indeed, when a plasmid containing the *MC066L* gene was transfected in human skin cell lines, many evidences supported the insertion of a selenocysteine residue at the UGA codon in the full-length protein, the functionality of the SECIS elements and the cellular antioxidant activity of the MC066L protein [175,239]. Remarkably, this viral selenoprotein has been shown to be protective for human keratinocytes against cytotoxic effects of UV-irradiation and hydrogen peroxides [175,239], suggesting an important function for the virus in defending itself against environmental stress and inflammation. How and when this selenoprotein is expressed in the context of viral infection remains poorly investigated. The first transcription map of the MCV genome was provided by the transcriptome sequencing (RNA-seq) of the RNAs synthesized in abortively infected cultured cells and human skin lesions [240]. These next generation sequencing experiments showed that MC066L mRNA was only detected in cutaneous lesions, but not in MRC-5, Huh7.5.1 and Vero cells infected *in vitro* by the MCV virus isolated from these same skin lesions. 

### 5.2. 2007: A Second Example of an Encoded Viral Selenoprotein in Fowlpox Virus Genome

Almost ten years later, another example of an encoded viral selenoprotein was reported in fowlpox viral genome [176], see Figure 5C,D. This was due to the increasing number of viral genome sequenced but also to the development of novel bioinformatic tools dedicated to the discovery of selenoprotein genes in newly sequenced genomes [79,80,81]. In this viral genome, a coding region homolog to the mammalian GPX4 gene was found, with an in-frame UGA codon, and a predicted SECIS element downstream of the UGA codon but this time within the open reading frame instead of being in the 3’UTR. This finding represented a great opportunity to investigate whether this putative viral SECIS or a canonical SECIS could function within the open reading frame. The authors demonstrated that mammalian cell lines supported the expression of selenoproteins with in-frame SECIS element from both viral and mammalian origin. This fowlpox SECIS element was the second example of a functional viral SECIS element with a structure being identical to the mammalian SECIS. Interestingly, in an evolutionary related virus, the canarypox virus (CPV), this gene has evolved in a Cys-containing GPX4 with a fossil SECIS element still present in the coding region (Figure 5C,D). The potential of this fossil SECIS to trigger recoding of an UGA codon in selenocysteine has not been investigated. It appears that there was a recent mutation of the selenocysteine into cysteine codon in canarypox virus, as it has happened multiple times during evolution of the selenoproteomes in *Eukarya*, *Bacteria* and *Archaea* [79,80]. Note that cysteine is encoded by UGC and UGU codons, and that a single mutation is able to change a selenocysteine to cysteine codon and vice-versa. The presence of a fossil SECIS element indicates that the GPX4 selenoprotein gene was first acquired from the host and recently converted to the Cys form.

The fact that at least two selenoproteins are encoded by viral genomes suggests that these proteins provide a substantial advantage for viruses. Similar to molluscum congatiosum GPX1, the fowlpox GPX4 may provide survival benefits for the virus. These two proteins are, so far, the only proven examples of genetically encoded viral selenoproteins.

### 5.3. Putative Selenoproteins in Other Viral Genomes

These two examples of selenoprotein gene snatching from eukaryotic genomes in the viral genomes of fowlpox and molluscum contagiosum viruses lead to the careful investigation for further examples of selenoprotein genes sequences with viral genomes. The first analysis searched for GPX modules within viral genomes where an in-frame UGA codon would be in an amino acid environment close to the catalytic site sequence of eukaryotic GPXs. Several candidates with sequence identities greater than 25% were found in the genomes of HIV-1, HIV-2, HCV, coxsackie virus B3 and measles viruses [183]. Despite these in silico data that GPX-related features are present in a number of RNA viruses, no RNA structure similar to the SECIS element can be evidenced. Additionally, no biochemical data demonstrated the expression of viral selenoproteins in any of these cases. It is possible that viruses have developed somewhat unique mechanisms for Sec insertion, as suggested in [195], but this remains purely hypothetical in the absence of further experimental proofs.

Perhaps, the most advanced study concerns a putative GPX protein coded in the third reading frame of the envelope (Env) gene of HIV-1 [241]. Indeed, it contains the typical catalytic triad selenocysteine (U), Glutamine (Q) and Tryptophan (W) and this putative HIV-GPX protein has been predicted to adopt the overall GPX fold, as deduced from computerized calculations [233]. In addition, it appears that the HIV-GPX gene is conserved in laboratory strains of HIV-1, as well as in long-term non-progressor isolates, but most of HIV isolates from patients with progressive disease presented deleterious mutations (mostly premature stop codons). In order to grasp the cellular function of this putative HIV-GPX in mammalian cells, the corresponding coding sequence has been fused to a mammalian SECIS element and transfected in mammalian cells [234]. The expression of the HIV-GPX seems to have an anti-apoptotic activity, by conferring cytoprotection against exogenous or endogenous ROS. Indeed, several viral proteins are known to induce apoptosis via redox-sensitive effects during HIV-1 viral cycle. Therefore, the presence of a HIV-GPX could be pertinent in the long-term non-progressor patients. Note that these experiments were performed before the emergence of the HAART.

Another putative viral selenoprotein gene has been reported in the -1 reading frame of the NS4 region of Japanese encephalitis virus (JEV). JEV belongs to the *Flaviviridae* family, which also includes dengue fever virus (DENV), yellow fever virus (YFV) and West Nile virus (WNV). The gene named NS4-fs encodes a potential 104 amino acid sequence with three predicted selenocysteine residues, i.e., three in frame UGA codons [191]. This putative selenoprotein displays 30.3% identity and 45.8% similarity with an aligned family of ferredoxin with cysteine instead of selenocysteine. Noteworthy, these three UGA codons are highly conserved, as they are present in all of the 15 full genomic JEV sequences analyzed. A 3D structure of the protein has been modeled [191] where the selenocysteine residues are proposed to maintain the conformation of the [Fe_2_S_2_] cluster center. Interestingly, ferredoxin usually acts as an electron transfer agent in biological redox reactions, and this may somehow be important for JEV infection or replication. Again, in this example, neither SECIS elements were found nor any biochemical evidence of selenoprotein expression was provided.

## 6. Conclusions

During viral infections, there are many ways that the host metabolism could be affected, leading to a dysregulation of redox homeostasis. The viral pathogens induce oxidative stress via the increase generation of ROS and the alteration of cellular ROS scavenging systems. As part of antioxidant defense, selenoproteins, such as GPXs, TXNRDs and those located in the ER, play an important role in controlling oxidative stress. Selenium deficiency creates a weakening of the defense against infectious diseases by reducing selenoprotein expression. However, nutritional status of the host can also lead to viral genome mutations from a benign or mildly pathogenic virus to a highly virulent one under oxidative stress that could further spread in hosts with adequate selenium intake. The molecular mechanism leading to the site-specific genome evolution of the virus toward more pathogenic strains awaits further experiments, especially to understand the implication of the selenoproteins. 

## Figures and Tables

**Table 1:** Scientific literature available on the link between selenium, selenoprotein and viral infections listed as a function of Baltimore classification.

Group	Genome Structure	Virus Family	Virus	Epidemiological Study	Epidemiological Intervention	*In Vitro* Study	*In Vivo* Study	Viral Selenoprot
I	Double-stranded DNA	*Herpesviridae*	Epstein-Barr virus (EBV)	↓ GPX activity associated with ↑ viral load [169]		CT = ombilical blood mononuclear cells SS = Se-rich rice extract Inhibits EBV mediated cell transformation [170]		[171]
			Herpes Simplex Virus 2 (HSV-2)		SS = Selenium aspartate + multisupplementation Faster healing, ↓ in viral load and ↑ in antiviral cytokines [172]	CT = Vero cells SS = Diphenyl diselenide ↓ infectivity [173]	AM = BALB/c Mice SS = Diphenyl diselenide ↓ histological damages and viral load ↑ levels of TNFalpha and IFNgamma [173]	
			Human Herpesvirus 3 (HHV3)		SS = Selenium aspartate + multisupplementation Faster healing, ↓ in viral load and ↑ in antiviral cytokines [172]			
			Cytomegalovirus (CMV)					[171]
			Infectious bovine rhinotracheitis (IBRV)		SS = Sodium selenite ↑ GPX activity after infection in Se group ↑ IgM after infection in Se group ↑ antibody titer after infection in Se group [174]			
		*Poxviridae*	Molluscum contagiosum virus (MCV)					[171,175]
			Fowlpox virus (FWPV)					[176]
		*Papovaviridae*	Human Papillomavirus (HPV)		SS = Selenium aspartate + multisupplementation Faster healing, ↓ in viral load and ↑ in antiviral cytokines [172,177]			
II	Single-stranded DNA	*Circoviridae*	Porcine Circovirus type 2 (PCV2)			CT = PK15 cells SS = selenite, selenocarrageenan and selenomethionine Selenomethionine inhibits replication of PCV2 via Gpx1 and oxidative stress [164,165] CT = PK15 cells SELENOS overexpression may ↓ viral replication via oxidative stress [166] CT = PK15 cells SS = selenizing astragalus polysaccharide and selenomethionine ↓ PCV2 replication through autophagy ↓ [167,168]	AM = KunMing Mice SS = Selenized yeast ↓ TNFalpha, viral load and histological damages [178]	
IV	Positive-sense single-stranded RNA	*Picornaviridae*	Coxsackievirus B3 (CVB3)				AM = C3H/HeJ Mice SS = Selenite Apparition of a more virulent strain after infection of a selenium or vitamin E deficient host due to viral mutations [98,99,110,111,179] AM = Gpx1-/- Mice Apparition of a more virulent strain after infection of GPX1 deficient mice [108] AM = C57Bl/6 Mice SS = not specified Co-infection with a retrovirus lead to a more virulent pathology Selenium supplementation reverse the effect [180] AM = Balb/c Mice Viral load is associated with selenium status in tissues [181] AM = Mice SS = commercial Se repleted ‘feedstuff’ Se deficient mice exhibit a higher mortality, histopathological pathogenicity and viral load [182]	[171,183]
			Coxsackievirus B4 (CVB5)					[183]
			Coxsackievirus B5 (CVB5)			CT = Vero Cells SS = selenite, selenate and selenomethionine Selenite ↓ viral replication via thiol interaction [184]		
			Live attenuated poliomyelitis vaccine		SS = Sodium selenite ↑ GPX activity after infection in Se group ↑ antiviral cytokines ↑ TH4 response Faster clearance Mutations in the viral particles [185]			
			Foot-and-mouth disease virus (FMDV)		SS = selenium enriched yeast ↑ GPX activity after infection in Se group ↓ DNA damage [186]			
V	Negative-sense single-stranded RNA	*Flaviviridae*	Hepatite C virus (HCV)	↓ Se in infected people [152,160,187] ↓ GPX activity in infected people [152] No variation in GPX activity with infection [145]	SS = Selenomethionnine ↑ GPX activity after infection in Se group No effect on viral load [188]			[183,189]
			West nile virus (WNV)			CT = Vero cells Selenium deficiency induces higher cell death and cytopathic effects but has no impacts on viral production [190]		
			Japanese encephalitis virus (JEV)					[191]
		*Bunyaviridae*	Hantaan virus (HTNV) or Seoul virus (SEOV)	↑ incidence of the infection with ↓ Se [192]		CT = HUVEC SS = sodium selenite ↓ viral replication with a low MOI [192]		
			Respiratory syncytial virus (RSV)	↓ Se in infected people [193]	SS = Sodium selenite Faster healing in Se group [194]			
		*Filoviridae*	Ebola virus (EBOV)					[195]
		*Orthomyxoviridae*	Influenza A/Bangkok/1/79 (H3N2)			CT = Differenciated human bronchial epithelial cells Se deficiency ↑ mucus production, influenza-induced apoptosis and modifies cytokine expression [122]	AM = C57Bl/6J SS = Selenite Deficiency leads to an ↑ in inflammation and pathology and an altered cytokine expression No changes in viral load [95] Apparition of a more virulent strain after infection of a selenium deficient host due to mutations [121] ↑ SOD activity in selenium deficient group [124] AM = transgenic mice carrying a mutant Sec-tRNA^[Ser]Sec^ No impact on lung pathology [123]	
			Influenza A/Puerto Rico/8/34 (H3N2)				AM = C57Bl/6J SS = Selenite Deficiency leads to altered immune response responsible of less death No change in viral load [125]	
			Influenza A (H1N1)	↓ Se in infected people [116,196] ↓ GPX activity in infected people [116]			AM = KunMing Mice SS = Selenite Reduces mortality, ↑ levels of TNFalpha and IFNgamma No change in viral load [197]	
			Avian influenza (H9N2)		SS sodium enriched yeast or sodium selenite ↓ viral shedding ↑ ISG expression and IFN [198]			
			Avian Influenza A/duck/Novosibirst56/05 (H5N1)			CT = RK, BHK21 and Vero E6 cells SS = nutrient mixture containing selenium ↓ viral replication in late stages [199]		
		*Paramyxoviridae*	Parainfluenza-3 (PI3)		SS = sodium selenite ↑ GPX activity after infection in Se group ↑ IgM after infection in Se group antibody titer after infection in Se group [200]			
			Human metapneumovirus (HMPV)	↓ Se in infected people [193]				
			Measles virus (MV)					[183]
VI	Single-stranded RNA with a DNA intermediate	*Retroviridae*	Human immuno-deficiency virus 1 (HIV-1)	↓ Se in infected people [153,187,201,202,203,204,205,206,207,208,209,210,211,212] No significant variation of Se level in infected people [213,214,215] Low selenium level associated with low CD4 count [153,203,206,207,211] Low selenium level associated with a higher progression to AIDS [201,202,203,204,206] Low selenium level associated with vaginal shedding of HIV [216] High selenium level associated with vaginal shedding of HIV [217] No significant variation of Se level in infected people treated with HAART [218] More skin desease in Se deficient HIV infected people [211]	No change in viral load SS = selenized yeast [137,139] SS = selenomethionine [219] SS = Sodium selenite [220] SS = not indicated [138,221] ↑ in CD4 count in Se group SS = selenized yeast yeast [137,139] SS = Sodium selenite [220] SS = not indicated [138,221] No change in CD4 count SS = selenomethionine [219,222] SS = not indicated [223] ↑ of viral shedding in Se group SS = selenomethionine [224] SS = not indicated [221] Se supplementation improves child survival if the mother is infected SS = selenomethionine [219] Se supplementation decreases diarrheal morbidity SS = selenomethionine [225]	CT = Jurkat and HeLa cells Infection or TAT expression ↓ some selenoproteins but ↑ low molecular mass selenocompounds [142,226] CT = ACH2, Jurkat, ESb-L, KK1, U1 cells and monocytes SS = selenite Prevents HIV transcription by TNF alpha mediated NFkappaB activiation in chronically infected cells [227,228,229] CT = SupT1 GPX1 overexpression ↑ viral replication and cytopathic effects and inversely [230] CT = U937, monocytes derived macrophages TXNRD1 negatively regulates TAT activity by targeting disulfides bonds [231]	In patients, a polymorphism a SELENOF is associated with a shorter time of progression to AIDS [232]	[195,233,234]
			Human immuno-deficiency virus 2 (HIV-2)					[183]
			Simian immuno-deficiency virus (SIV)	↓ Se in infected monkeys [235]		CT = CEM and Jurkat cells Infection leads to a ↓ in selenoprotein expression and an ↑ in low molecular mass selenocompounds TAT transfection leads to a ↓ in GPX and SELENOF but an ↑ in TXNRD1 expression [235]		
			Murine Leukemia virus (MuLV)		SS = Sodium selenite ↑ GPX activity after infection in Se group [236]			[171]
VII	Double-stranded DNA with a single stranded RNA intermediate	*Hepadnaviridae*	Hepatitis B (HBV)	↓ Se in infected people [158,160] {↑ Se associated with less hepatic damages Abediankenari, 2011 #4334}	SS = selenized table salt or selenized yeast Lower cancer induced by HBV incidence [162]	CT = HepG2 and HuH7 SS = sodium selenite Suppresses HBV replication, transcription and protein expression [161]		[171]

SS, type of selenium supplementation used in the study; CT, cell type used for the study; AM, animal model used for the study; Se, selenium; ↓ decrease, ↑ increase.
